# Early response to COVID-19 in the  Philippines

**DOI:** 10.5365/wpsar.2020.11.1.014

**Published:** 2021-02-05

**Authors:** Arianna Maever L. Amit, Veincent Christian F. Pepito, Manuel M. Dayrit

**Affiliations:** a College of Medicine, University of the Philippines Manila, Manila, Philippines.; b School of Medicine and Public Health, Ateneo de Manila University, Pasig City, Philippines.

## Abstract

Low- and middle-income countries (LMICs) with weak health systems are especially vulnerable during the COVID-19 pandemic. In this paper, we describe the challenges and early response of the Philippine Government, focusing on travel restrictions, community interventions, risk communication and testing, from 30 January 2020 when the first case was reported, to 21 March 2020. Our narrative provides a better understanding of the specific limitations of the Philippines and other LMICs, which could serve as basis for future action to improve national strategies for current and future public health outbreaks and emergencies.

## The Philippine health system and the threat of public health emergencies

Despite improvements during the past decade, the Philippines continues to face challenges in responding to public health emergencies because of poorly distributed resources and capacity. The Philippines has 10 hospital beds and six physicians per 10 000 people. (*1*, *2*) and only about 2335 critical care beds nationwide. (*3*) The available resources are concentrated in urban areas, and rural areas have only one physician for populations up to 20 000 people and only one bed for a population of 1000. (*4*) Disease surveillance capacity is also unevenly distributed among regions and provinces. The primary care system comprises health centres and community health workers, but these are generally ill-equipped and poorly resourced, with limited surge capacity, as evidenced by lack of laboratory testing capacity, limited equipment and medical supplies, and lack of personal protective equipment for health workers in both primary care units and hospitals. (*5*) Local government disaster preparedness plans are designed for natural disasters and not for epidemics.

Inadequate, poorly distributed resources and capacity nationally and subnationally have made it difficult to respond adequately to public health emergencies in the past, as in the case of typhoon Haiyan in 2013. (*6*) The typhoon affected 13.3 million people, overwhelming the Government’s capacity to mobilize human and financial resources rapidly to affected areas. (*7*) Failure to deliver basic needs and health services resulted in disease outbreaks, including a community outbreak of gastroenteritis. (*8*) Access to care has improved in recent years due to an increase in the number of private hospital beds; (*5*) however, improvements in private sector facilities mainly benefit people who can afford them, in both urban and rural areas.

In this paper, we describe the challenges and early response of the Philippine Government, focusing on travel restrictions, community interventions, risk communication and testing, from 30 January 2020 when the first case was reported, to 21 March 2020.

## Early response to COVID-19

### Travel restrictions

Travel restrictions in the Philippines were imposed as early as 28 January, before the first confirmed case was reported on 30 January (**Fig. 1a**). (*9*) After the first few COVID-19 cases and deaths, the Government conducted contact tracing and imposed additional travel restrictions, (*10*) with arrivals from restricted countries subject to 14-day quarantine and testing. While travel restrictions in the early phase of the COVID-19 response prevented spread of the disease by potentially infected people, travellers from countries not on the list of restricted countries were not subject to the same screening and quarantine protocols. The restrictions were successful in delaying the spread of the disease only briefly, as the number of confirmed cases increased in the weeks that followed. (*11*) **Fig. 1b** shows all interventions, including travel restrictions undertaken before 6 March, when the Government declared the occurrence of community spread, and after 11 March, when WHO declared COVID-19 a pandemic.

**Figure 1a:**
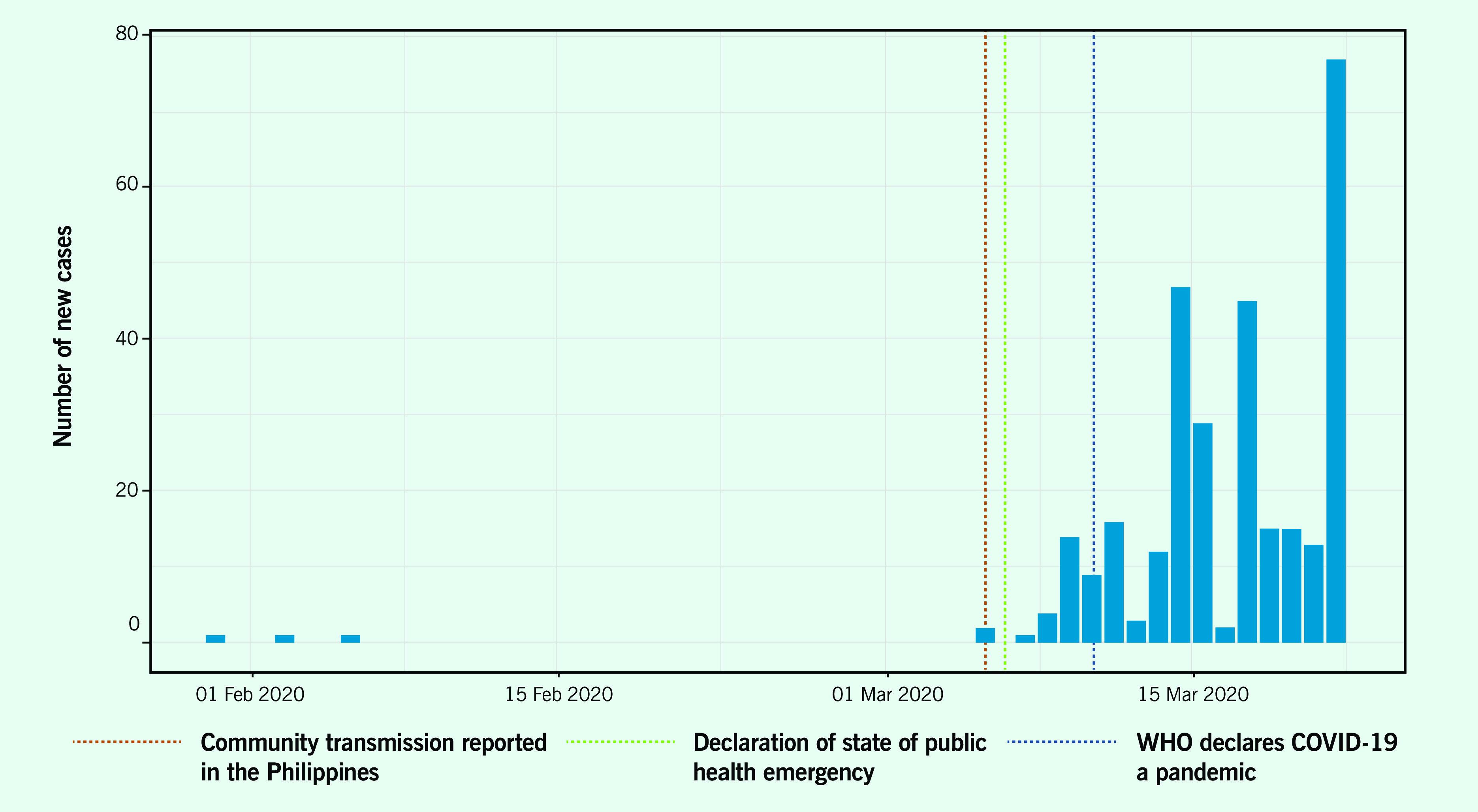
New cases of COVID-19 in the Philippines, 30 January–21 March 2020

[**insert** Figure 1a]

**Figure 1b:**
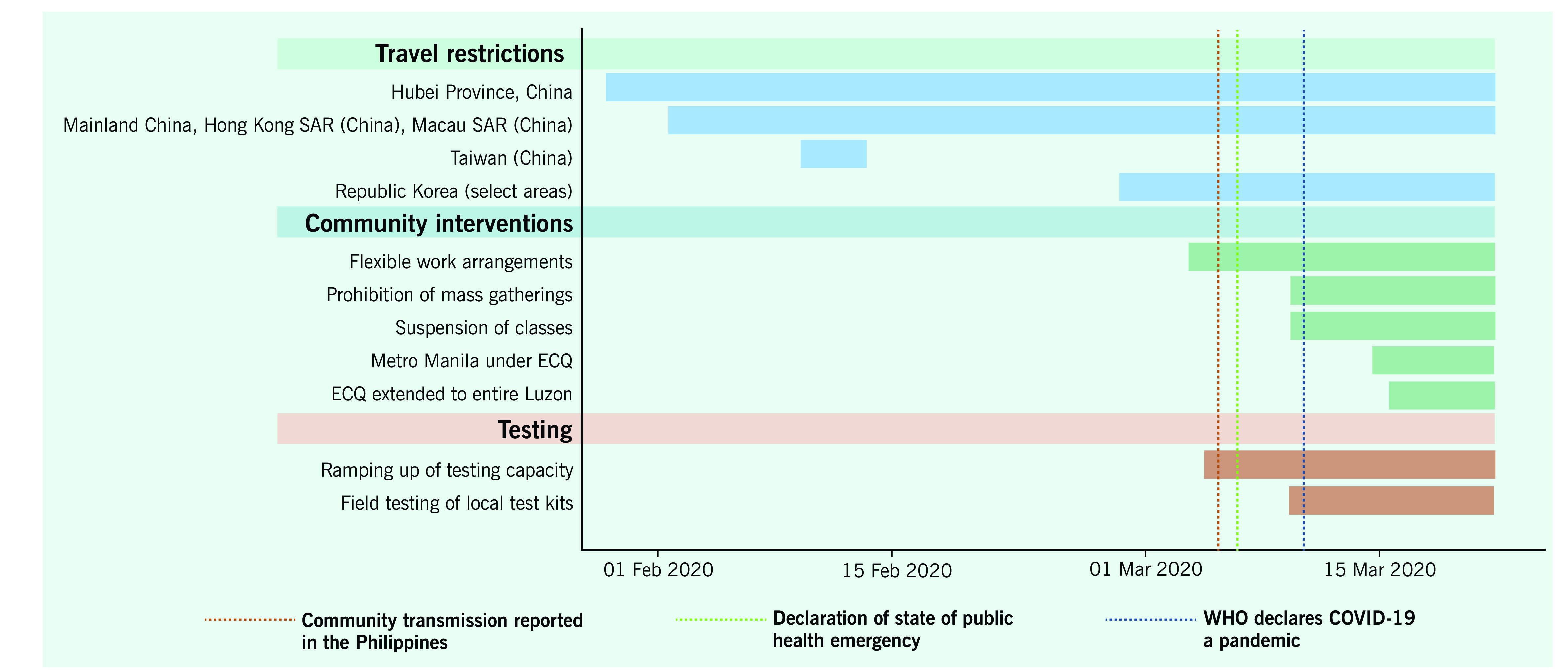
Timeline of key events and developments in the Philippines, 30 January–21 March 2020

[**insert** Figure 1b]

### Community interventions

**Figure 2:**
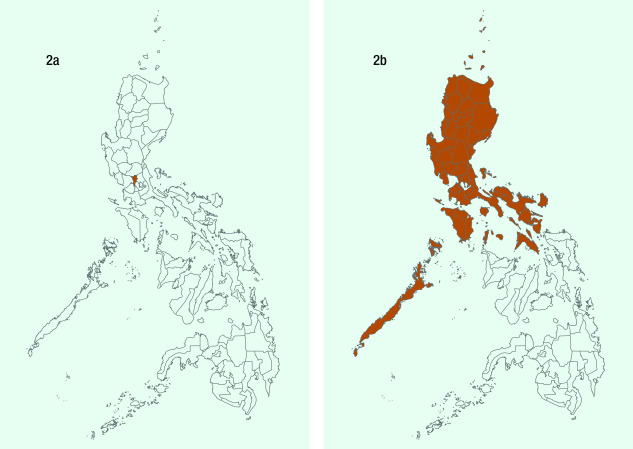
Provinces placed under enhanced community quarantine (ECQ). (2a) The Government declared ECQ in Metro Manila effective 15 March 2020; (2b) The Government declared ECQ on the entire island of Luzon effective 17 March 2020.

The Government declared “enhanced community quarantine” (ECQ) for Metro Manila between 15 March and 14 April (**Fig. 2a**), which was subsequently extended to the whole island of Luzon (**Fig. 2b**). The quarantine consisted of: strict home quarantine in all households, physical distancing, suspension of classes and introduction of work from home, closure of public transport and non-essential business establishments, prohibition of mass gatherings and non-essential public events, regulation of the provision of food and essential health services, curfews and bans on sale of liquor and a heightened presence of uniformed personnel to enforce the quarantine procedures. (*12*) ECQ – an unprecedented move in the country’s history – was modelled on the lockdown in Hubei, China, which was reported to have slowed disease transmission. (*13*) Region-wide disease control interventions, such as quarantining of the entire Luzon island, were challenging to implement because of their scale and social and economic impacts, but they were deemed necessary to “flatten the curve” so that health systems were not overwhelmed. (*14*) While the lockdown implemented by the Government applied only to the island of Luzon, local governments in other parts of the country followed this example and also locked down. The ECQ gave the country the opportunity to mobilize resources and organize its pandemic response, which was especially important in a country with poorly distributed, scarce resources and capacity.

[**insert** Figure 2]

### Risk communication

The Government strengthened and implemented national risk communication plans to provide information on the new disease. The Government conducted daily press briefings, sponsored health-related television and Internet advertisements and circulated infographics on social media. Misinformation and conspiracy theories about COVID-19 were nevertheless a challenge for a population that spends more than 10 hours a day on the Internet. (*15*, *16*) These spread quickly and became increasingly difficult to correct. Furthermore, the Government’s messages did not reach all households, despite access to health services and information, resulting in limited knowledge of preventive practices, except for hand-washing. (*17*)

### Testing

Testing is key to controlling the pandemic but was done on a small scale in the Philippines. As of 19 March, fewer than 1200 individuals had been tested, (*11*) as only the Research Institute for Tropical Medicine located in Metro Manila performed tests and assisted subnational reference laboratories in testing. (*18*) No positivity rates for RT–PCR tests were reported until early April 2020. Because of the limited capacity for testing at the start of the pandemic, the Department of Health imposed strict protocols to ration testing resources while ramping up testing capacity. Most tests were conducted for individuals in urban areas, where the incidence was highest. (*19*)

## Conclusions

At the start of the COVID-19 pandemic, the country’s initial response lacked organizational preparedness to counter the public health threat. The Philippines’ disease surveillance system could conduct contact tracing, but this was overwhelmed in the early phases of outbreak response. Similarly, in February, only one laboratory could conduct reverse transcriptase polymerase chain reaction (RT–PCR) testing, so the country could not rapidly deploy extensive laboratory testing for infected cases. In addition, the primary care system of the Philippines did not serve as a primary line of defence, as people went straight to hospitals in urban areas, overwhelming critical care capacity in the early stages of the COVID-19 pandemic.

In response to the early phase of the pandemic, the Government of the Philippines implemented travel restrictions, community quarantine, risk communication and testing; however, the slow ramping up of capacities particularly on testing contributed to unbridled disease transmission. By 15 October, the number of confirmed cases had exponentially grown to 340 000 of which 13.8% were deemed active. (*11*) The lack of pandemic preparedness had left the country poorly defended against the new virus and its devastating effects. Investing diligently and consistently in pandemic preparedness, surveillance and testing capacity in particular is a lesson that the Philippines and other LMICs should learn from COVID-19.
